# Switchable phase and polarization singular beams generation using dielectric metasurfaces

**DOI:** 10.1038/s41598-017-07217-5

**Published:** 2017-07-28

**Authors:** Yanliang He, Ying Li, Junmin Liu, Xiaoke Zhang, Yao Cai, Yu Chen, Shuqing Chen, Dianyuan Fan

**Affiliations:** International Collaborative Laboratory of 2D Materials for Optoelectronics Science and Technology, and Key Laboratory of Optoelectronic Devices and Systems of Ministry of Education and Guangdong Province, Shenzhen University, Shenzhen, 518060 P. R. China

## Abstract

Singular beams which possess helical phase wavefront or spatially inhomogeneous polarization provide new freedom for optical field manipulation. However, conventional schemes to produce the singular beams have difficulty in realizing the flexible switch between different singular beams. In this work, we have experimentally demonstrated the capability of dielectric metasurfaces to generate three types of singular beams and switch between them at working wavelength of *1550* *nm*. We have shown vortex beam and cylindrical vector beam generation with single metasurface and cylindrical vector vortex beam generation with two cascaded metasurfaces. Moreover, experimental demonstration on switching cylindrical vector beam into vortex beam has also been done by combining one quarter-wave plate and a Glan laser polarizer. The experimental results match well with the analysis from the Jones matrix calculations. The average conversion efficiency of cylindrical vector beam to vortex beam was estimated to be 47.7%, which was about 2.3% lower than the theoretical prediction.

## Introduction

Singular beam, which usually has a doughnut-like intensity profile in the transverse plane, has been widely studied for various applications due to its unique optical properties^1, 2^. Generally, there are two types of singular beams, namely phase singularity and polarization singularity. Optical beam with phase singularity, also known as vortex beam (VB), is characterized by the helical phase-front^1^. Polarization singularity, which has spatially inhomogeneous polarization distribution across its cross-section, is usually referred to cylindrical vector beam (CVB)^2^. Both VB and CVB has attracted much attention due to their unique optical properties and have shown exciting perspective in various applications ranging from optical manipulation to imaging and optical communication etc^3–13^. Especially for optical communication, it is believed that the phase singularity and vector singularity can be used as new multiplexing/modulation dimension to improve the communication capacity and capacity density^7, 8, 11–13^. More interestingly, the spatially inhomogeneous polarization and helical phase wavefront can coexist in one light beam and thus form cylindrical vector vortex (CVV) beam, which can provide additional degrees of freedom for beam manipulation^14, 15^.

The efficient generation of different singular beams and the flexibility in switching between them are the fundamental challenges that need to be solved. Currently, sundry devices and methods have been proposed to generate singular beams^16–29^. However, it is found that only spatial light modulator (SLM) and radial polarization converter^18, 20, 24–28^ could be used to produce all of them (CVB, VB and CVV beam). It is well known that the SLM is much more expensive than other ordinary optical elements. Moreover, CVV beam generation with SLM involves complex light path. On the contrary, the radial polarization converters are compact and the light path for the singular beams generation is simple. Among all the radial polarization converters, optical metasurfaces with micro-nano structure are most attractive due to their exceptional properties, such as high damage threshold, simple structure and high stability^30^, which make the dielectric metasurfaces become a leading way for singular beam creation. Researchers have already studied the generation of single VB, CVB and CVV beam by using metasurface, however, little attention has been put in the beam transformation and flexible switching between singular beams. Hence, it is meaningful to propose a novel approach of generating different singular light beams with one setup.

In this work, we have experimentally demonstrated the generation of CVBs, VBs and CVV beams and the flexible switching between them based on dielectric metasurfaces which are fabricated by writing space-variant grooves in a silica glass. For the potential applications in optical communications, such as optical route, optical information processing, the experiment and Jones matrix calculations were carried out at working wavelength of 1550 *nm*. In order to further demonstrate the flexibility in switching between these singular beams, a system comprising of a quarter-wave plate and a Glan laser polarizer was well-designed for transforming CVB into linearly polarized VB. The average conversion efficiency of CVB to VB was estimated to be approximate 47.7%, which is only 2.3% lower than the theoretical prediction. Moreover, we also show operation of switching VB into CVV beam by inserting a half-wave plate and another metasurface at the end position. The average conversion efficiency of VB to CVV beam can be as high as 82.4%.

## Results

### Theoretical analysis of metasurface

The metasurfaces used in this article were similar to q-plate in theory, which is fabricated by etching space-variant grooves on a fused silica sample using a femtosecond laser. The grooves are expected to result in a space-variant birefringence. We can achieve desired polarization distribution by adjusting the local orientation and geometrical parameter of the grooves. The direction of optical axis can be specified as follow:

1 $$\varphi (r,\theta )=q\theta +{\varphi }_{0}$$

where (*r*, *θ*) is the polar coordinate representation, *θ* = arctan(*y*/*x*) is the azimuthal angle, *ϕ*_0_ is the initial direction of axis, namely, the value of *ϕ*, when the value of *θ* is set up to zero. *q* is a constant indicating the spatial rotation ratio of the optical axis. In this paper, discussion is carried out on the basis of *ϕ*_0_ = 0.

Next we derive the transformation Jones matrix of space-variant metasurfaces. The Jones matrix of a wave-plate with fast axis in the horizontal direction can be written as:

2 $${M}_{wp}=[\begin{array}{cc}{e}^{i\delta /2} & 0\\ 0 & {e}^{-i\delta /2}\end{array}]$$

Here *δ* is the phase retardation of wave-plate. For the metasurface we used, its Jones matrix can be represented as ref. 24:

3 $$M(\varphi )=R(-\varphi ){M}_{wp}R(\varphi )$$

where

4 $$R(\varphi )=[\begin{array}{cc}\cos \,\varphi  & \sin \,\varphi \\ -\sin \,\varphi  & \cos \,\varphi \end{array}]$$

Here, we put the Eqs (2) and (4) into Eq. (3), the position-dependent Jones matrix *M*(*ϕ*) can be expanded into:

5 $$M(\varphi )=[\begin{array}{cc}\cos (\delta /2)-i\,\cos (2\varphi )\sin (\delta /2) & -i\,\sin (2\varphi )\sin (\delta /2)\\ -i\,\sin (2\varphi )\sin (\delta /2) & \cos (\delta /2)+i\,\cos (2\varphi )\sin (\delta /2)\end{array}]$$

The used metasurfaces have a homogeneous birefringent phase retardation of *π* (half-wave) and the inhomogeneous orientation of the fast optical axis is parallel to the transverse plane. The Jones matrix *M*(*ϕ*) can be further simplified as:

6 $${M}_{hw}(\varphi )=[\begin{array}{cc}\cos (2\varphi ) & \sin (2\varphi )\\ \sin (2\varphi ) & -\cos (2\varphi )\end{array}]=[\begin{array}{cc}\cos (2q\theta ) & \sin (2q\theta )\\ \sin (2q\theta ) & -\cos (2q\theta )\end{array}]$$

## Experimental results

### Generating VB based on metasurface

**Figure 1:**
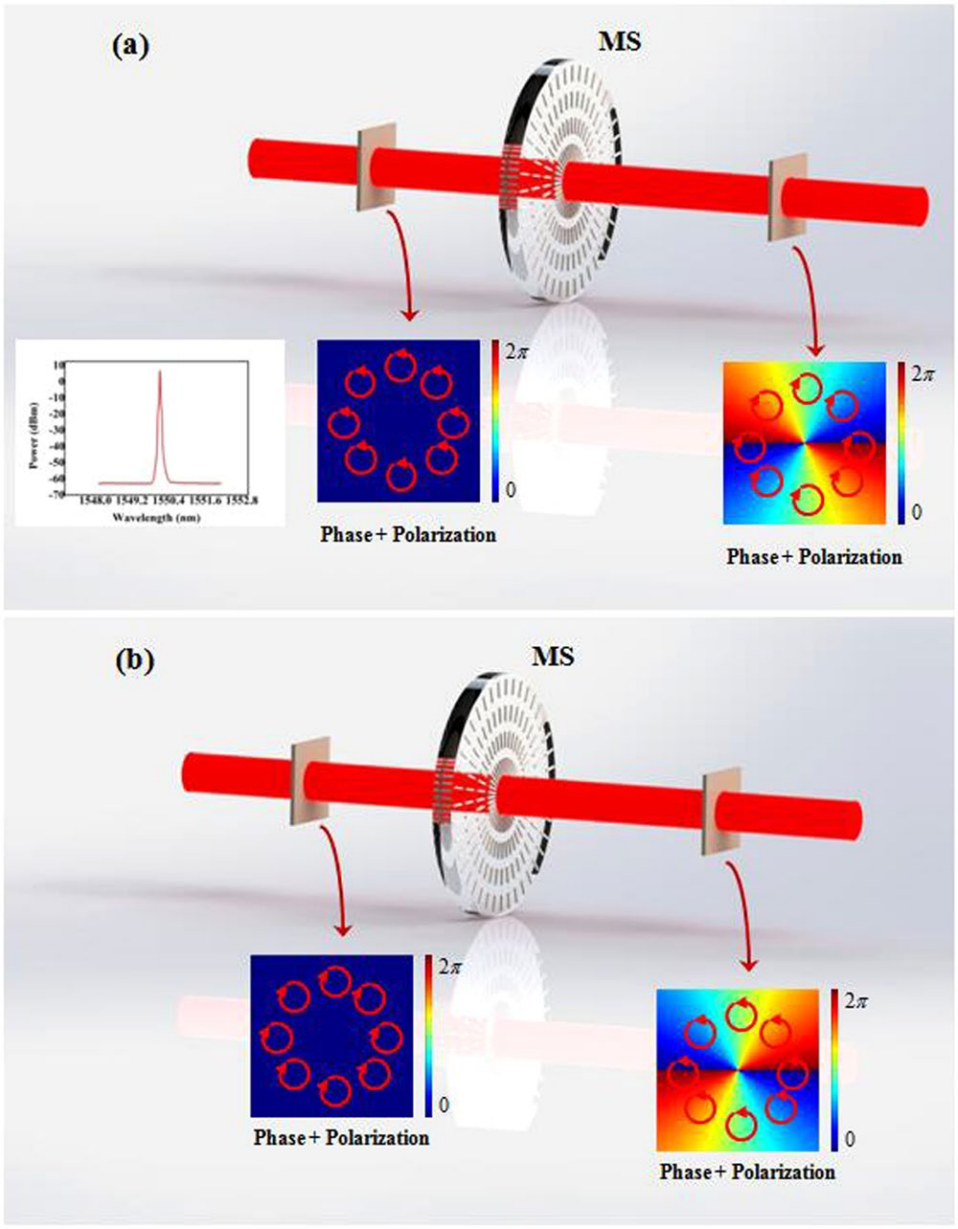
The conventional optical system applied to generate VBs. MS: metasurface; (**a**) A LCP (RCP) light beam illuminates the metasurface to generate VB, the optical spectrum is showed in the lower left corner. (**b**) A RCP light beam illuminates the metasurface to generate VB. Insets show the phase and polarization patterns in each step of this system.

Left circular-handed polarized or right-handed circular polarized light, described by the Jones electric-field vector ${E}_{in}={E}_{0}[1,{\sigma }_{\pm }i]$, here E_0_ is the amplitude, σ_+_ = +1 for the left-handed circular polarization (LCP) and σ_−_ = −1 for the right-handed circular polarization (RCP), respectively. After passing through the metasurface, as showed in Fig. 1(a and b), the input wave will be transformed into the following output wave

7 $${E}_{out}={M}_{hw}{E}_{in}={M}_{hw}{E}_{0}[\begin{array}{c}1\\ {\sigma }_{\pm }i\end{array}]={E}_{0}{e}^{\pm i2q\theta }[\begin{array}{c}1\\ {\sigma }_{\mp }i\end{array}]$$

where *q* is a constant indicating the spatial rotation ratio of the metasurface. From Eq. (7) we can see that the half-wave metasurface can invert the handedness of the incident circular polarized light. Meanwhile, the output wave has acquired a helical phase and the topological charge equal to 2*q*. The sign of the topological charge is positive for LCP, but negative for RCP.

**Figure 2:**
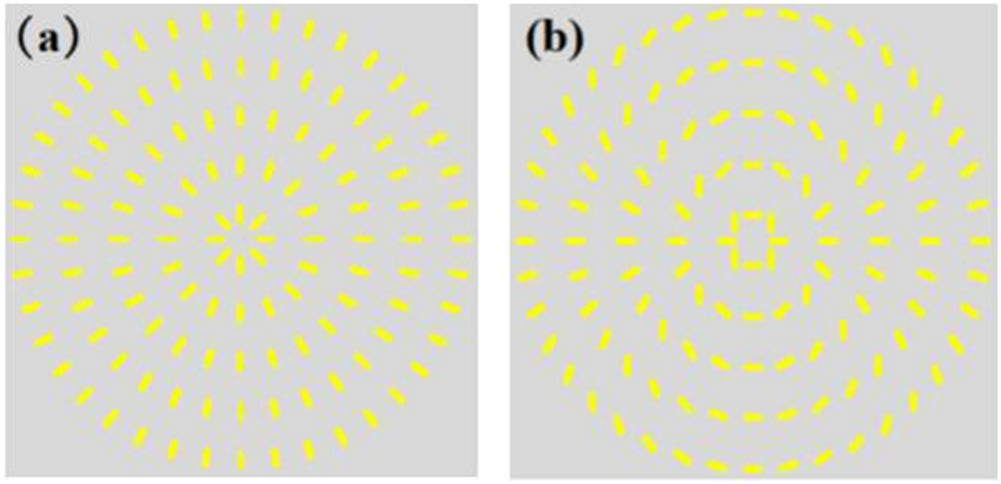
The optical axis distributions of the metasurfaces. Theoretical optical axis distributions of the metasurface (**a**) *q* = 1, (**b**) *q* = 2.

**Figure 3:**
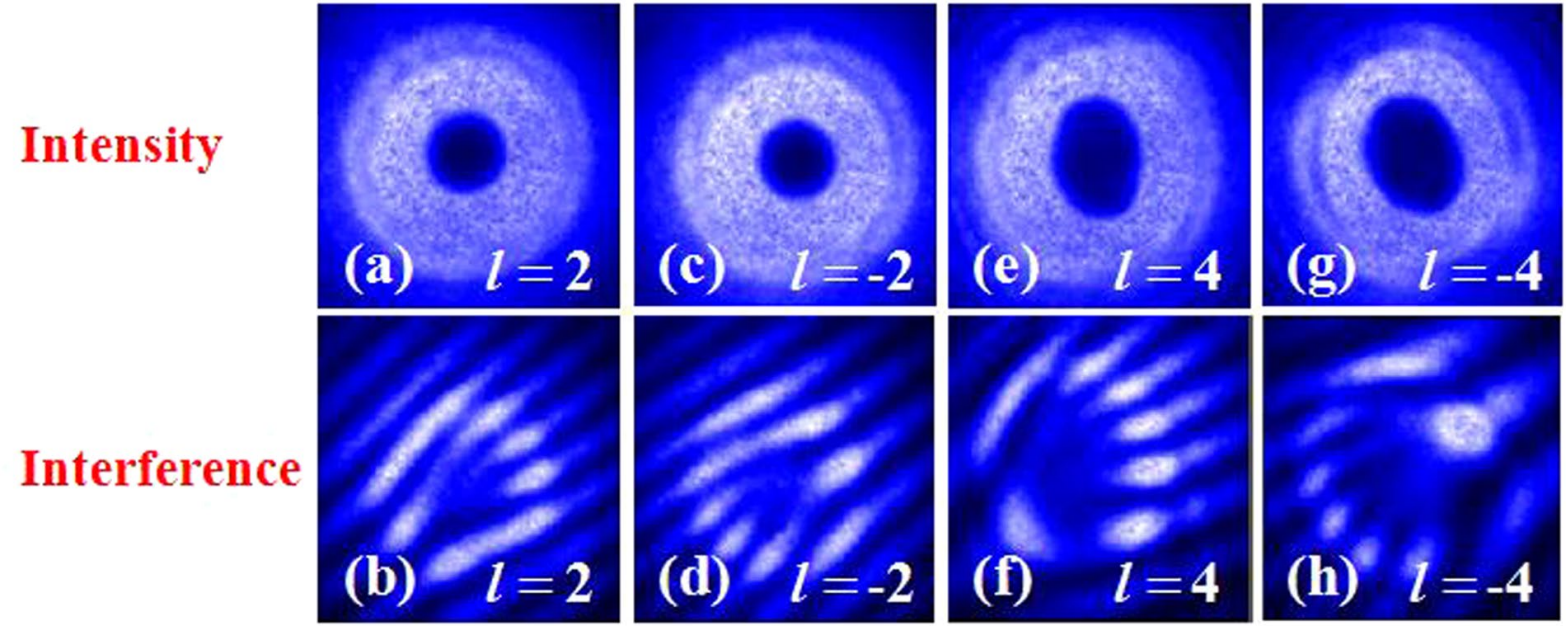
The intensity profiles and interference patterns of VBs obtained by using metasurface. Four different topological charges (*l*), +2, −2, +4 and −4 are chosed. The first row shows the intensity profiles and the second row shows the interference pattern.

The q-value of the adopted metasurfaces were *q* = 1 and *q* = 2, respectively. As shown in Fig. 2, the optical axis distribution of metasurfaces was presented. Figure 3 shows experimental results corresponding to this situation. In this experiment, firstly, we used a fundamental Gaussian monochromatic beam as the input beam, which was produced by a continue wave (CW) laser working at the wavelength 1550 *nm*. The optical spectrum is showed in the lower left corner of Fig. 1(a). Through using combination of Glan laser polarizer (GL) and quarter-wave plate (QWP) to generate LCP or RCP. The general intensity profiles of the output light beams (VB) present a doughnut shape. From Fig. 3(a) we can see that, with the increase of the topological charge, the radius of the doughnut-shape ring is increasing. And the radius of the doughnut-shape ring for the positive topological charge and negative topological charge is same. In order to measure the wavefront shape and the topological charge of the VB, the beam was utilized to interfere with a plane wave. The process of interference was carried out by combing VB with a plane wave by using a combiner. As shown in Fig. 3(b), the number of dislocated interference fringes is equivalent to the topological charge. And when the topological charge is a positive integer, the direction of the fork fringes is oblique upward. Conversely, the direction of the fork fringes is oblique downward.

**Figure 4:**
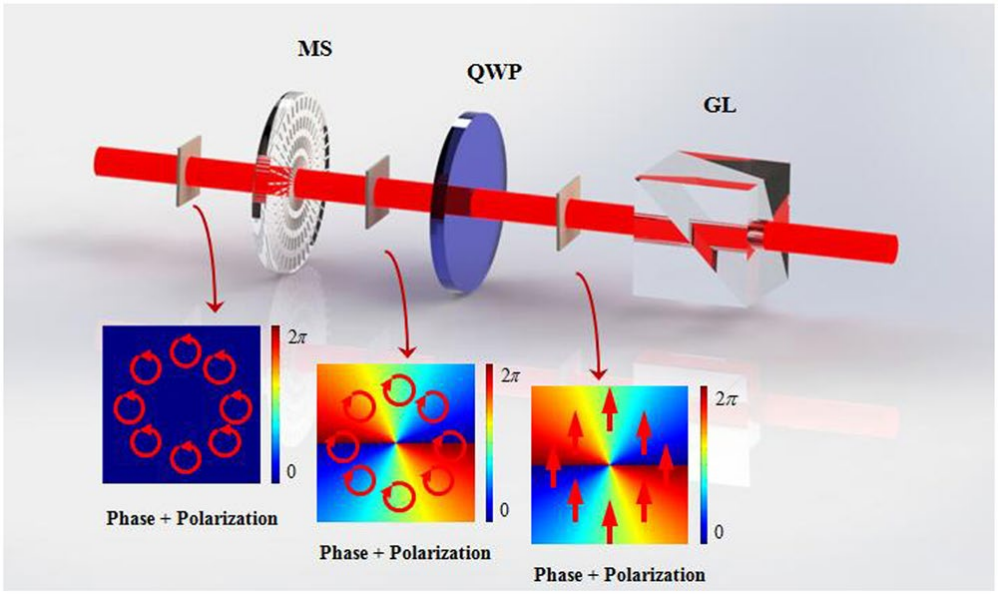
The optical system applied to measure the handedness of the obtained VBs. MS: metasurface; QWP: quarter-wave plate; GL: Glan laser polarizer. The metasurface generate a LCP (RCP) VB, a quarter-wave plate convert the LCP (RCP) to horizontal (vertical) polarization, and a Glan laser polarizer is used to measure the polarization state. Insets show the phase and polarization patterns in each step of this system.

**Figure 5:**
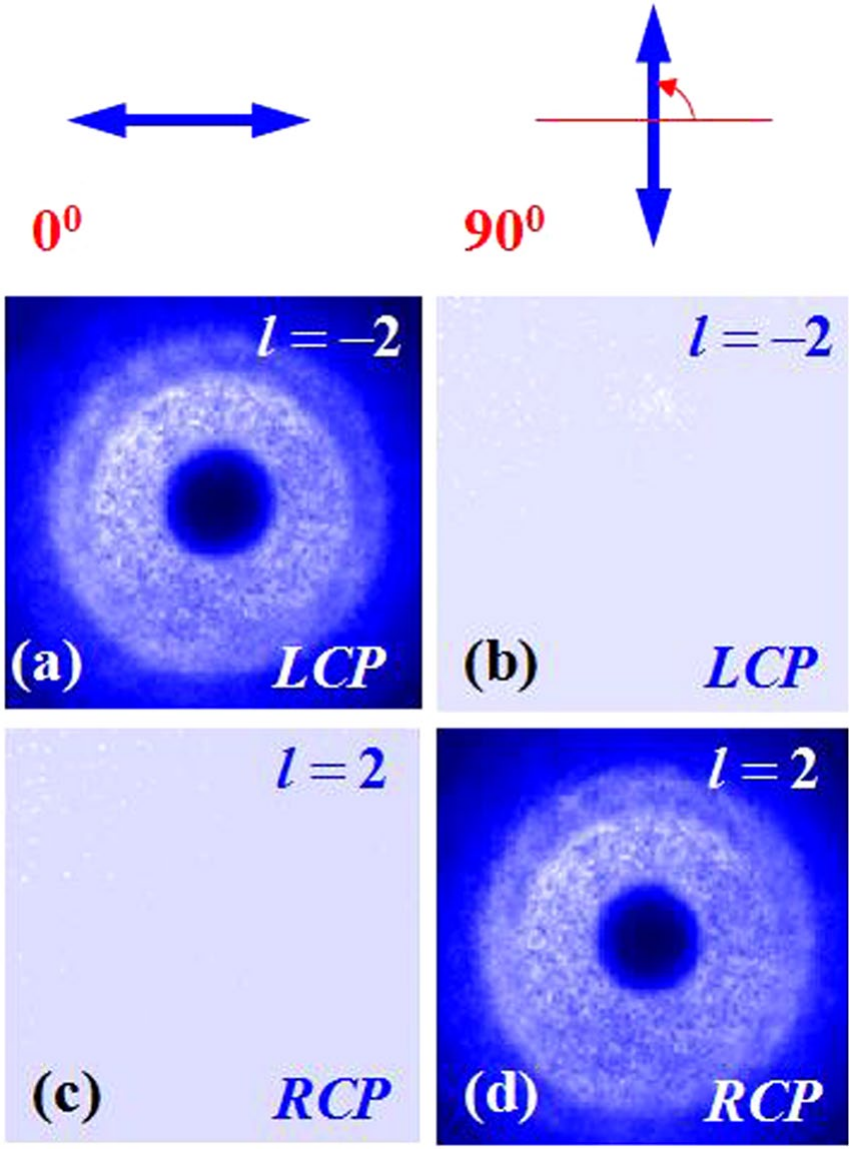
Measured intensity profiles by using the combinations of a QWP and a GL. The second row and third row show the measurement results of LCP (topological charges (*l*) was −2) and RCP (topological charges (*l*) was +2) VBs, respectively. Two axis orientations of polarizer 0° and 90° (blue arrows in the first row) are selected in experiment.

To further investigate the handedness of the output light beam after metasurface, an experimental scheme was carried out as showed in Fig. 4. After the RCP (LCP) Gaussian beam passing through the metasurface with the q-value of 1, the LCP (RCP) VB was obtained. It is known that a QWP whose fast axis was rotated to +45° can be utilized to transform the LCP (RCP) into horizontal (vertical) polarization. The combination of QWP whose fast axis was rotated to +45° and a GL was used for measuring the handedness of the VB. When the GL was rotated to 0°, the measured intensity was remained indicated that the polarization state was RCP, and no intensity indicates the polarization state was LCP. When the GL was rotated to 90°, the measured intensity was remained that the polarization state was LCP, and no intensity indicated the polarization state is RCP. Experimental results are shown in Fig. 5.

### Generating CVB based on metasurface

**Figure 6:**
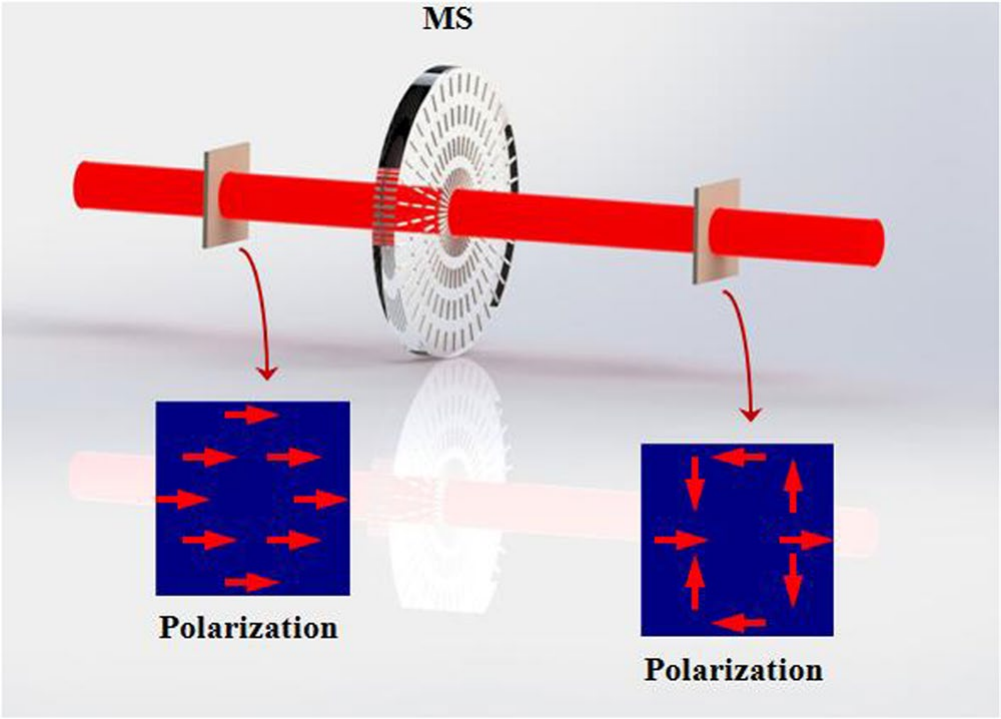
The conventional optical system applied to generate CVB. A horizontal (vertical) linear polarized light beam can be used to generate radial (azimuthal) CVB by using metasurface. Insets show the polarization patterns in each step of this system.

A horizontal linear polarized plane wave, which described by the Jones electric-field vector E_in_ = E_0_[1, 0], pass through the metasurface with a q-value of *q*_1_, as shown in Fig. 6, will be transformed into the following output wave

8 $${E}_{out}={M}_{hw}{E}_{in}={E}_{0}[\begin{array}{cc}\cos (2\varphi ) & \sin (2\varphi )\\ \sin (2\varphi ) & -\cos (2\varphi )\end{array}]\,[\begin{array}{c}1\\ 0\end{array}]={E}_{0}[\begin{array}{c}\cos (2{q}_{1}\theta )\\ \sin (2{q}_{1}\theta )\end{array}]$$

From Eq. (8) we can see that the half-wave metasurface can invert the homogeneous polarization state of the incident light into inhomogeneous polarization state, namely, the metasurface can be used to generate the CVB. Meanwhile, the output wave’s polarization order equals to 2*q*_1_.

**Figure 7:**
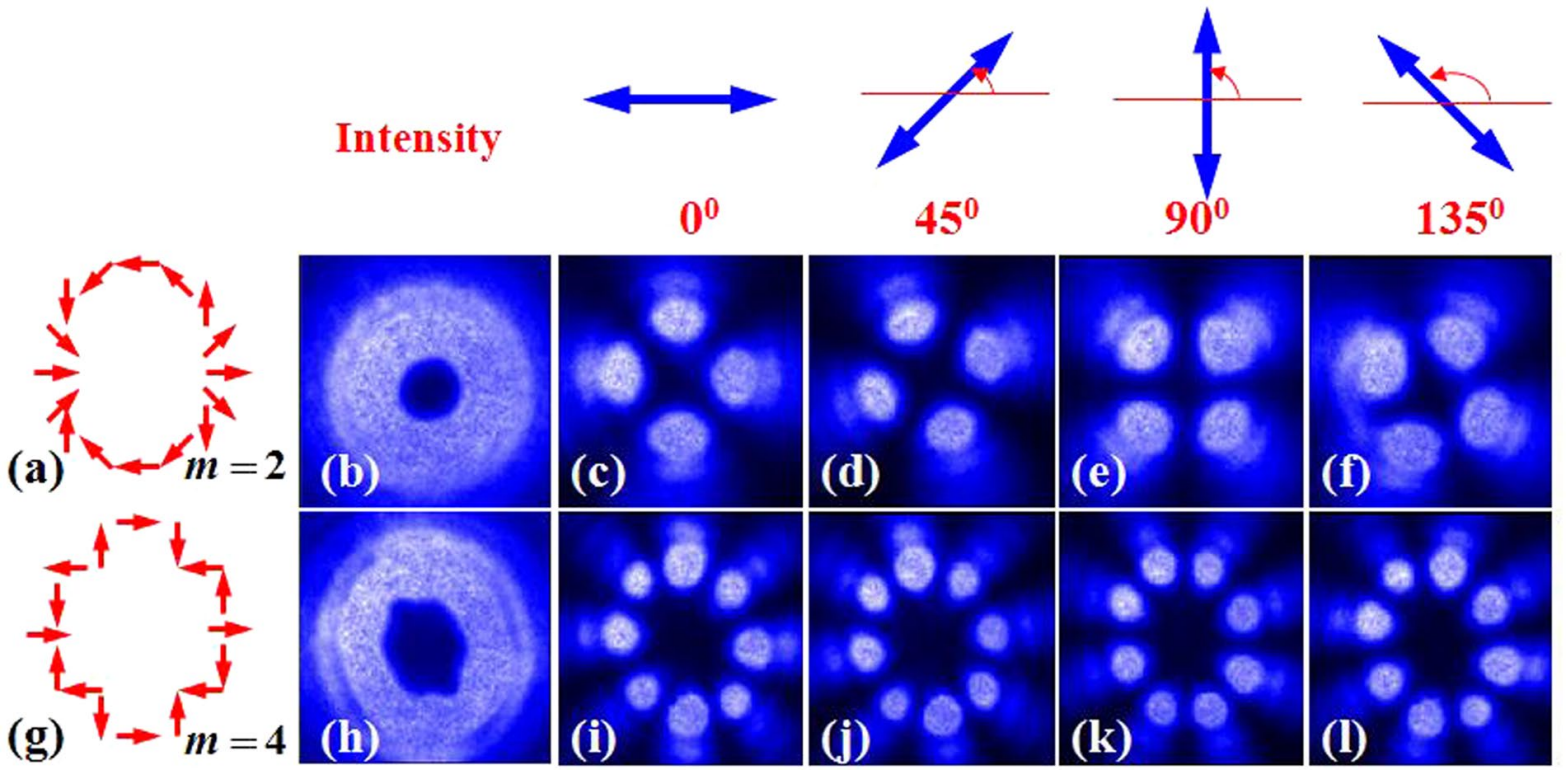
The intensity profiles and analyzed patterns of CVBs obtained by using metasurface. Two different polarization orders *m* = 2 and *m* = 4 are displayed from up to down. The first column shows the schematic polarization distributions (red arrow diagrams), of which the experimental results are presented in each row respectively. Different axis orientations of polarizer 0°, 45°, 90° and 135° (blue arrows in the first row) are selected in this experiment.

Figure 6 shows experimental results corresponding to this situation. A GL was be used to generate horizontal polarized Gaussian light beam. The q-value of the adopted metasurfaces were *q* = 1 and *q* = 2, respectively. The typical intensity profiles of the output light beams (CVB) also present a doughnut shape. In order to measure the inhomogeneous polarization state of the vector beam, the intensity profiles of the CVBs were measured with a GL located in front of the CCD. Experimental results are showed in Fig. 7.

### Generating CVV beam based on metasurface

**Figure 8:**
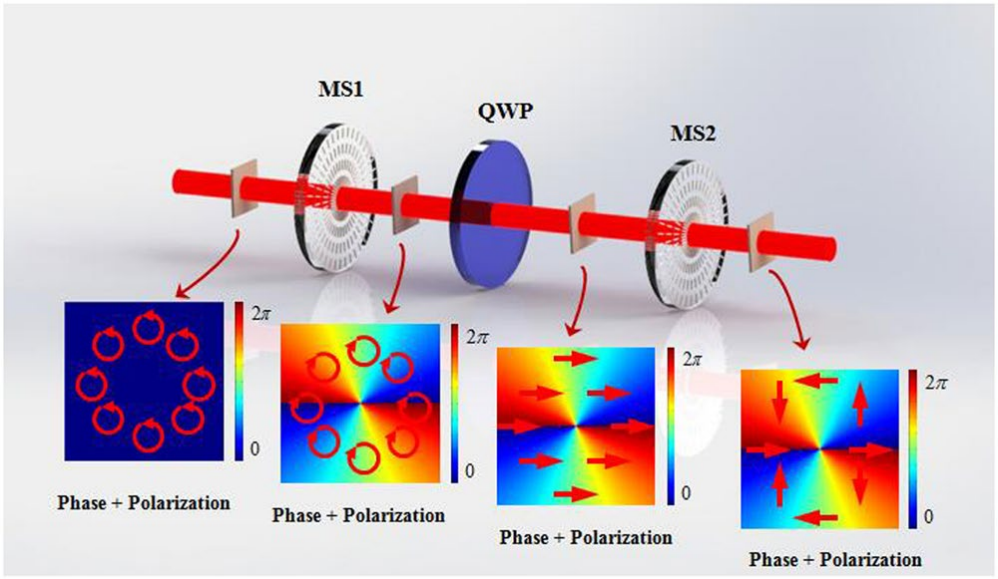
The optical system applied to generate CVV beam. MS: metasurface; QWP: quarter-wave plate. The first metasurface was used to generate RCP VB. The QWP was used for converting the RCP to horizontal linear polarization. The second metasurface was used to generate CVV beam by incident the horizontal linear polarization VB. Insets show the polarization and phase patterns in each step of this system.

From Eq. (7), we can see that a horizontal linear polarized vortex beam with topological charge of *l* can be simplified as ${E}_{0}{e}^{\pm il\theta }[1,0]$, after passing through the metasurface with the q-value of *q*_1_, as shown in Fig. 8, the output wave can be written as

9 $${E}_{out}={E}_{0}{e}^{\pm il\theta }[\begin{array}{cc}\cos (2{q}_{1}\theta ) & \sin (2{q}_{1}\theta )\\ \sin (2{q}_{1}\theta ) & -\cos (2{q}_{1}\theta )\end{array}]\,[\begin{array}{c}1\\ 0\end{array}]={E}_{0}{e}^{\pm il\theta }[\begin{array}{c}\cos (2{q}_{1}\theta )\\ \sin (2{q}_{1}\theta )\end{array}]$$

As you may have noticed from Eq. (9), the output wave can carry not only helical phase, but also inhomogeneous polarization state. The topological charge is ±*l* and the polarization order is 2*q*_1_.This kind of light beam is so-called CVV beam. In this experiment, the first metasurface (MS1) was used to generate RCP vortex beam. After passing through the QWP which was rotated to −45°, the horizontal linear polarized vortex beam was obtained. Another metasurface (MS2) was put behind the QWP, the system for generating CVV beam was erected.

**Figure 9:**
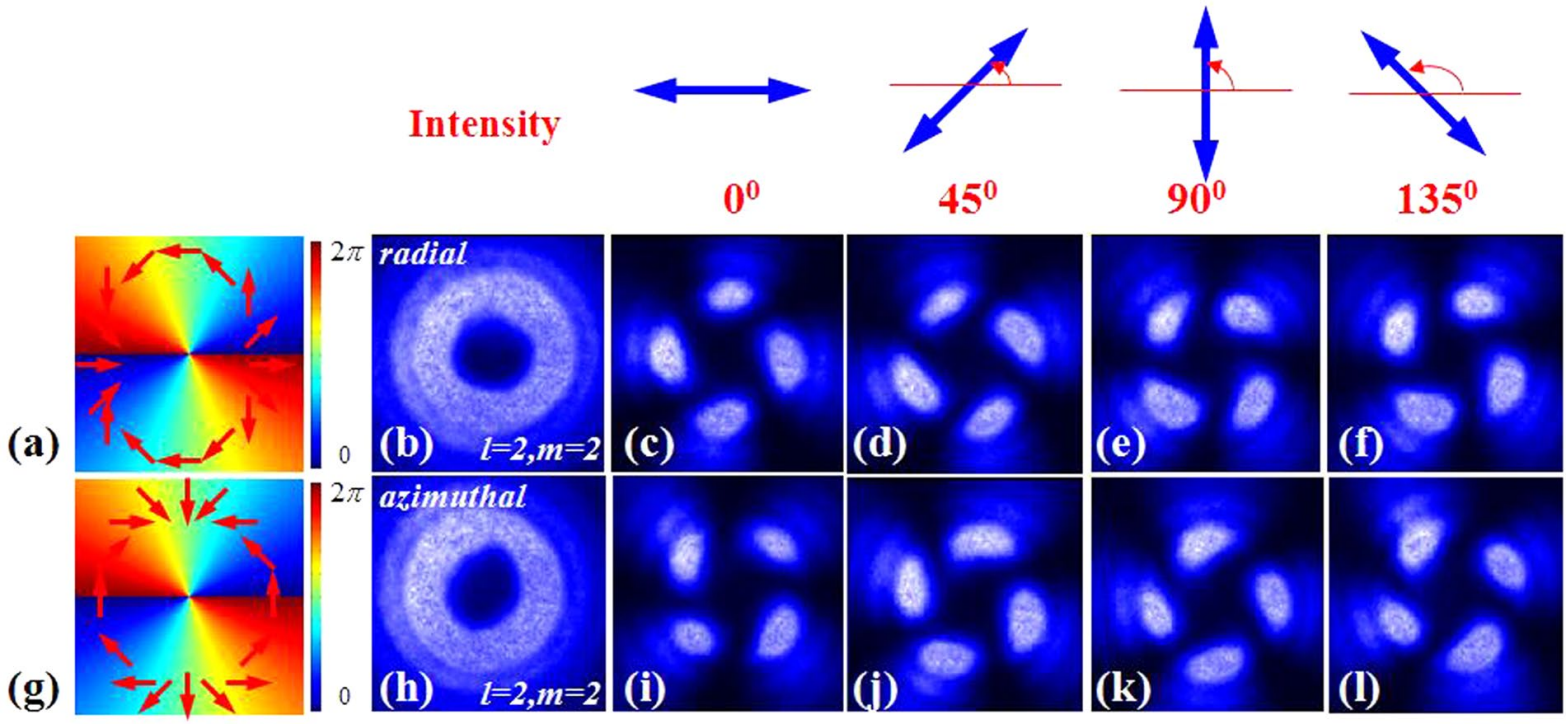
Experimentally generated CVV beams by cascading two metasurface and a QWP. Two different cylindrical vector polarizations of radial and azimuthal are displayed from up to down. The polarization order (*m*) and the topological charge (*l*) both were 2. The first column shows the schematic polarization distributions (red arrow diagrams) and phase distributions (periodically changing color background), of which the experimental results are presented in each row respectively. Different axis orientations of polarizer 0°, 45°, 90^0^ and 135^0^ (blue arrows in the first row) were selected in this experiment.

Experimental results are showed in Fig. 9. The q-value of the first metasurfaces and the second one both were 1. The typical intensity profiles of the output light beams (CVV beam) also present a doughnut shape. Intensity profiles of the CVV beams were also measured with a GL located in front of the CCD. By rotating the fast axis of the QWP to −45° or +45°, we have succeeded in obtaining radial and azimuthal CVV beams, respectively.

### Switching CVB into VB and VB into CVV beam in one system

**Figure 10:**
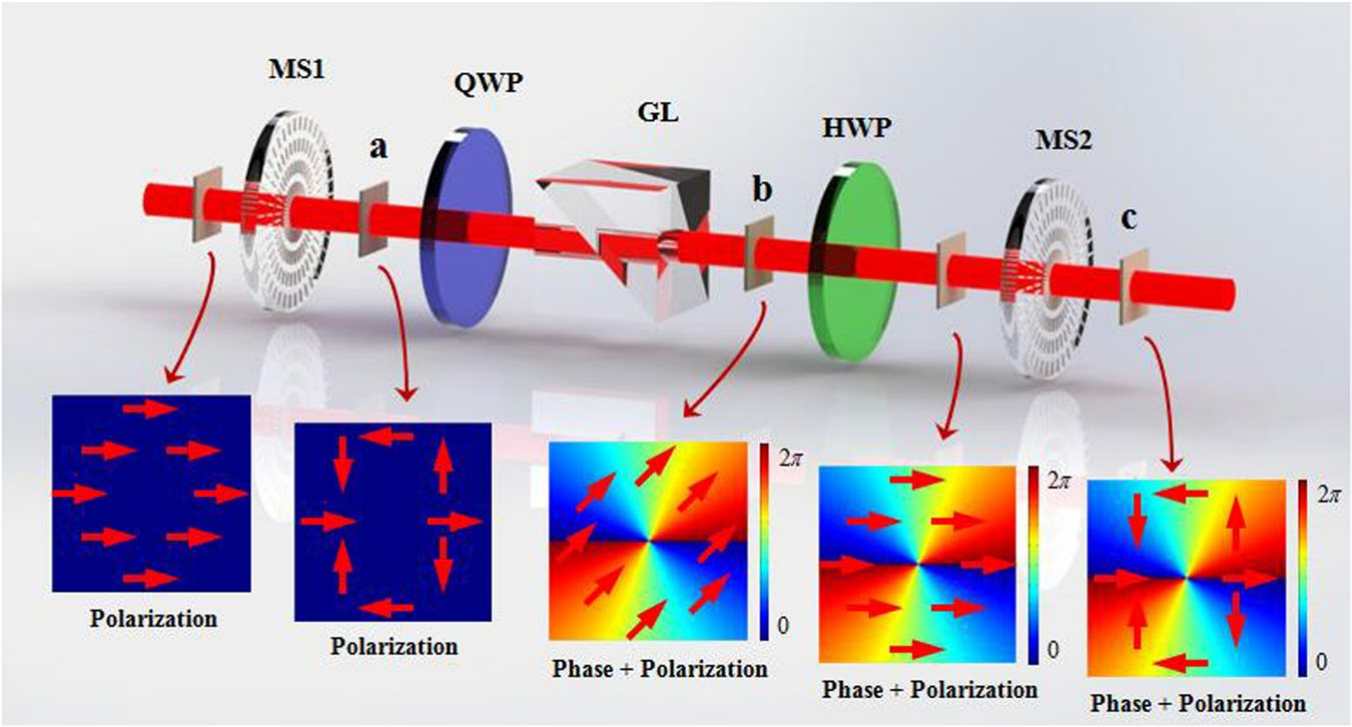
The optical system applied to switch CVB into VB and obtain VB, CVB and CVV beam in one system. MS: metasurface; QWP: quarter-wave plate; GL: Glan laser polarizer; HWP: half-wave plate. The first metasurface was used to generate CVB. Combinations of QWP, GL and HWP are used to convert the CVB to horizontal linear polarized VB. The second metasurface was used to generate CVV beam by incident the horizontal linear polarization VB. Insets show the polarization and phase patterns in each step of this system.

The experimental system is showed in Fig. 10. This system was consisting of metasurface, GL, HWP and QWP. A CVB can be obtained at point *a* with a horizontal linear polarized Gaussian beam passing through a metasurface whose q-value was *q*_1_. The CVB can be written as

10 $${E}_{out1}={E}_{0}[\begin{array}{c}\cos (2{q}_{1}\theta )\\ \sin (2{q}_{1}\theta )\end{array}]$$

After passing through a QWP with the fast axis was parallel to the horizontal axis, and an analyzer which was rotated to +45^0^, the output wave at point *b* can be written as

11 $${E}_{out2}={E}_{0}[\begin{array}{cc}{\cos }^{2}(\pi /4) & 1/2\cdot \,\sin (\pi /2)\\ 1/2\cdot \,\sin (\pi /2) & {\sin }^{2}(\pi /4)\end{array}]\,[\begin{array}{cc}{e}^{i\pi /4} & 0\\ 0 & {e}^{-i\pi /4}\end{array}]\,[\begin{array}{c}\cos (2{q}_{1}\theta )\\ \sin (2{q}_{1}\theta )\end{array}]={{E}_{0}}^{^{\prime} }{e}^{-i2{q}_{1}\theta }[\begin{array}{c}1\\ 1\end{array}]$$

A HWP was used to rotate the polarization state to horizontal. So, after another metasurface with the q-value of *q*_2_ was inserted behind the HWP, the output wave at point *c* can be written as

12 $${E}_{out3}={E}_{0}{e}^{-i2{q}_{1}\theta }[\begin{array}{c}\cos (2{q}_{2}\theta )\\ \sin (2{q}_{2}\theta )\end{array}]$$

From Eq. (12) we can see that the generated CVV beam’s polarization order was 2*q*_1_ and topological charge was −2*q*_1_.

**Figure 11:**
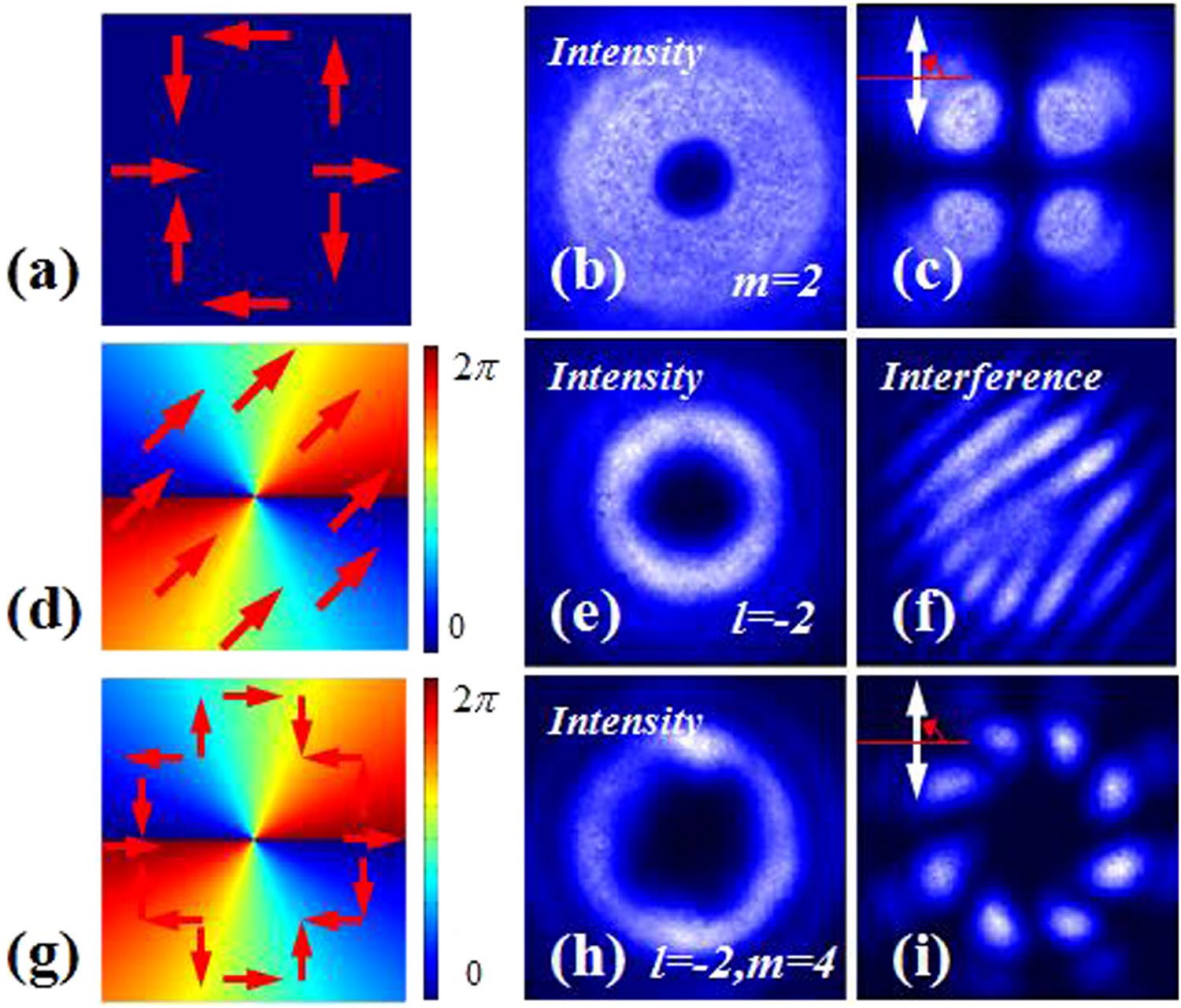
The intensity profiles and analyzed patterns of the singularity beams obtained by the above system. The polarization order (*m*) of the CVB (at point *a*) is 2, the topological charge (*l*) of the converted VB (at point *b*) is −2, the polarization order and the topological charge of the generated CVV beam (at point *c*) is 4 and −2, respectively. The first column shows the schematic polarization distributions (red arrow diagrams) and phase distributions (periodically changing color background), of which the experimental results are presented in each row respectively. The axis orientations of polarizer 90^0^ (white arrows in the top left corner) was selected in the analysis of CVB and CVV beam. The VB is analyzed by interfering with a plane wave.

**Figure 12:**
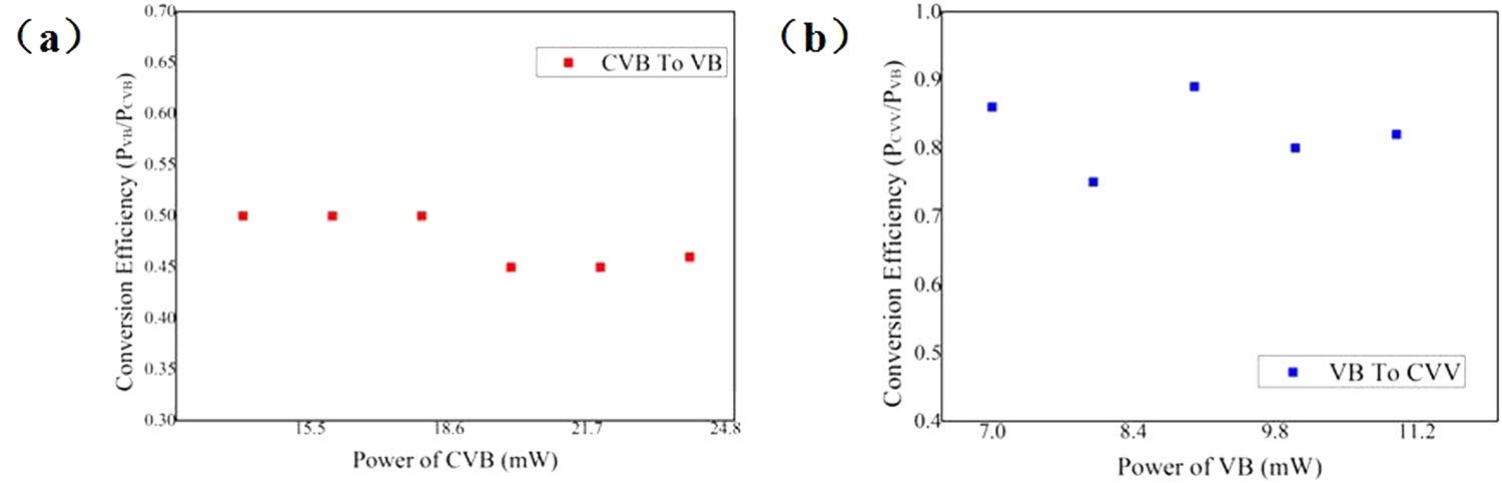
The experimental conversion efficiencies of CVB to VB and VB to CVV beam. (**a**) The conversion efficiency of CVB to VB (the ratios of the VB’s power P_VB_ to the CVB’s power P_CVB_). (**b**) The conversion efficiency of VB to CVV beam (the ratios of the CVV’s power P_CVV_ to the VB’s power P_VB_).

In this experiment, the q-value of the first metasurfaces was 1 and the second one was 2, respectively. Experimental results are showed in Fig. 11. Analyzed with a polarizer, we can ensure that the CVB (polarization order *m* =2 ) was generated at the point *a* and the CVV beam (polarization order *m* = 4 and topological charge *l* = −2) was obtained at point *c*. By interfering with a plane wave, we can ensure that the VB (topological charge *l* = −2) was generated at point *b*. It was demonstrated that the combination of QWP and GL can be definitely used to switch CVB into linear polarized VB. And we can easily transform VB into CVV beam with a HWP was used to adjust the polarization state of the VB and another metasurface located at the end of the light path. Theoretical calculation predicted that the conversion efficiency of CVB to VB (the ratios of the VB’s power P_VB_ to the CVB’s power P_CVB_) was 50%. As shown in Fig. 12(a), with the increasing of CVB’s power, the conversion efficiencies of CVB to VB were kept almost 50%. The difference between the average conversion efficiency and the theoretical prediction was about 2.3%. Owing to the transmission of the metasurface was greater than 75%, the average conversion efficiency of VB to CVV beam (the ratio of the CVV’s power P_CVV_ to the VB’s power P_VB_) was about 82.4%, as shown in Fig. 12(b).

## Discussion

In conclusion, we have experimentally demonstrated the generation of CVBs, VBs and CVV beams and the flexible switching between them by using metasurface. Experimental results show that the single metasurface can not only be used to transfer a LCP (RCP) Gaussian beam into RCP (LCP) vortex beam, but also be applied to generate CVB with a linear polarized Gaussian beam. Meanwhile, by cascading two metasurfaces and a HWP inserted between them can be used to generate CVV beam, the polarization order and topological charge can be changed by replacing the metasurfaces with another two. It is also experimentally proved that the combination of QWP and GL can be used to switch CVB into linear polarized VB. The average conversion efficiency of CVB to VB was estimated to be 47.7%. And the difference between the average conversion efficiency and theoretical value was about 2.3%. With a HWP being used to adjust the polarization state of the VB and another metasurface located at the end of the light path, this scheme could used to switch VB into CVV beam. The average conversion efficiency of VB to CVV beam can reach 82.4%. We believe that these results will catch the attention of both the optical communication field and optics field.

## Methods

### Experimental measurements

The schematic of experimental setups are shown in Figs 1, 4, 6 and 10. In Figs 1 and 4 the metasurface was used to generate VB, and the combination of GL and QWP was used to prepare the polarization state of incidence. In Fig. 4 QWP was used to convert the polarization state of VB into linear polarization, and GL was used to measure the polarization. In Fig. 6 the metasurface is used to generate CVB, and GL was used to prepare the polarization state of incidence. In Fig. 8 the combination of the first metasurface and QWP was used to generate linear polarized VB, and the second metasurface was used to give vector polarization distribution to the linear polarized VB. In Fig. 10 the first metasurface was used to produce CVB, then the combination of QWP, GL and HWP was used to switch CVB into linear polarized VB, and the second metasurface was used to generate CVV beam. All the measurement of the VB was realized by interfering with a plane wave and the polarization order was analyzed by using GL. All the metasurfaces are fabricated using femtosecond laser writing in fused silicon glass boards and their operating wavelength are 1550 *nm*; efficient diameter is 6 *mm* (Workshop of Photonics). In these experiments, a continue wave laser operating in 1550 *nm* (15 *dBm*, Keysight N7714A) served as the optical source. A wavefront sensor (PHASICS SID4-NIR) was applied to record all the intensities. A power meter (Newport 1919-R) was applied to measure all the power of light beams.
